# Acute arterial ischemic stroke in children: single-center experience

**DOI:** 10.1055/s-0045-1809661

**Published:** 2025-06-25

**Authors:** Muharrem Bostancı, Arzu Ekici, Cengiz Havalı

**Affiliations:** 1 University of Health Sciences, Bursa Yuksek Ihtisas Training and Research Hospital, Department of Pediatrics, Bursa, Turkey.

**Keywords:** Cerebral Infarction, Magnetic Resonance Imaging, BE-FAST, Child

## Abstract

**Background:**

Arterial ischemic stroke (AIS) is an increasingly common disorder in childhood that causes severe mortality and morbidity.

**Objective:**

To emphasize the importance of early diagnosis and treatment of AIS cases, as well as the early initiation of physical therapy.

**Methods:**

We retrospectively reviewed the medical records of 23 patients aged 1 month to 18 years, who were admitted with acute neurological complaints and diagnosed radiologically with AIS at the Bursa Yüksek İhtisas Training and Research Hospital, a tertiary healthcare facility, between January 2016 and June 2020.

**Results:**

Neurological deficit was the most common (12 patients; 52%) complaint, followed by seizure in 5 patients (21%), facial paralysis in 5 patients (21%), and visual impairment in 3 patients (13%). We performed brain computed tomography (CT) scans in 12 (52%) patients at the first application, and infarction was detected in 8 (66%). Infarction was in the cerebrum in 19 (82%) patients. Mutations of the *methylenetetrahydrofolate reductase* ( *MTHFR* ; A1298C and 677C > T) gene were found most frequently. Cardiac anomalies were detected in 7 (30%) patients, and 2 patients had ventriculoperitoneal shunts.

**Conclusion:**

Arterial ischemic stroke presents very different clinical findings, such as hiccups, anisocoria, and upward gaze paralysis. We wanted to emphasize the importance of cranial CT scanning because sometimes MRI is not always easily performed in children. Concomitant conditions, such as intracranial operation, ventriculoperitoneal shunt, and hypochondroplasia, may increase the risk of stroke.

## INTRODUCTION

Arterial ischemic stroke (AIS) is an increasingly common disorder in childhood that causes severe mortality and morbidity. 1 A population-based study 2 found an incidence of AIS of 2.4 per 100 thousand inhabitants and a mortality rate of 4%. More than half of the patients with AIS develop permanent neurological damage and cognitive and psychiatric disorders, and one-third develop epilepsy, which also causes negative economic and psychiatric consequences for both families and society. 3 It is important to establish an early diagnosis and start physical therapy as soon as possible. 4 However, the diagnosis of AIS is delayed due to the presence of nonspecific and age-dependent varying symptoms, the frequency of other diseases compared with stroke, imaging methods that are not easily performed in children, and due to the etiology and risk factors, which are heterogeneous. 5

There is a wide variety of risk factors in the etiology of AIS in children. The most important risk factor is congenital or acquired heart disease, which is also detected in one-third of the cases. 6 Other risk factors are sickle cell anemia, autoimmune or inflammatory conditions, recent chickenpox, moyamoya disease, and cerebral arteriopathies. 7 In adults, early thrombolytic treatments contribute significantly to the healing process of the disease. 8 Treatments such as intravenous recombinant tissue plasminogen activator (rt-PA) and thrombectomy are rarely applied in pediatric cases; there is not enough evidence regarding their safety and effectiveness. 9

The current study aims to evaluate the clinical signs, risk factors, neuroimaging findings, treatments, and short-term outcomes of cases of AIS to contribute to the development of early-diagnosis and treatment strategies.

## METHODS

We retrospectively evaluated the medical records of 23 patients aged 1 month to 18 years, who were admitted with acute neurological complaints and diagnosed radiologically with AIS at the Bursa Yüksek İhtisas Training and Research Hospital (a tertiary healthcare facility) between January 2016 and June 2020. The institutional Ethics Committee approved the study protocol (under decision number 2011-KAEK-25 2019/09–19).

We excluded children aged < 1 month and patients who were diagnosed with hemorrhagic stroke and sinus vein thrombosis. The entire history of each patient was recorded, including stroke onset, clinical manifestations, and other systemic illnesses. We assessed age, gender, presentation complaints, computed tomography (CT) scans, magnetic resonance imaging (MRI) scans, diffusion MRI scans, MR angiography (MRA) findings, the time of patient admission, the time until the diagnosis of stroke confirmed by any imaging method, the risk factors, and the treatments. All of our patients' complaints were evaluated through the Face Drooping, Arm Weakness, Speech Difficulty, and Time to Call Emergency (FAST) test, recommended by the American Stroke Association, and the Balance, Eyes–Face Drooping, Arm Weakness, Speech Difficulty, and Time to Call Emergency (BE-FAST) scale. The results of the following tests were recorded: blood count, C-reactive protein (CRP), serum electrolytes, triglycerides, total cholesterol, prothrombin time, activated thromboplastin time, protein C, protein S, homocysteine, electrocardiography, and echocardiography. The modified Trial of ORG 10172 in Acute Stroke Treatment (TOAST) classification was used to categorize all patients based on their radiological, laboratory, and clinical data, and the patients were classified into 6 groups as follows: 10 11 large vessel atherosclerosis; cardioembolic; small vessel disease; other determined etiology; multiple probable/possible causes; and undetermined etiology.

### Statistical analysis

For the statistical analysis, we used the IBM SPSS Statistics for Windows (IBM Corp.) software, version 22.0,. Descriptive statistical data were expressed as percentages (%). The normal distribution of the data was determined according to the Kolmogorov-Smirnov test. The results were expressed as mean, standard deviation, and 95%CI values for the numerical data showing a normal distribution. Median, maximum, minimum, and quartile values were used to express the numerical values and the categorical data that did not show normal distribution. An independent group *t* -test was used in the comparative analysis of the normally-distributed numerical data. The Mann-Whitney U test was used to analyze the categorical and the numerical data that did not show normal distribution. Values of *p* < 0.05 were considered significant.

## RESULTS

A total of 23 patients diagnosed with AIS were included in the present study. Of these, 65% ( *n* = 15) were male subjects, while 35% ( *n* = 8) were female patients (male-to-female ratio: 2:1). Their average age at presentation was of 4.9 years (range: 1.5 months–17.5 years). In total, 6 (26%) patients were in the adolescent age group. Among the children, the average age of the girls was of 5.7 years, and that of the boys was of 6.5 years. We found no statistical difference in terms of age and gender distribution ( *p* > 0.05). The most common complaint was of neurological deficit, in 12 (55%) patients, followed by seizure in 5 patients (21%), facial paralysis in 5 patients (21%), and visual impairment in 3 patients (13%; anisocoria in 1 patient, upward gaze restriction and diplopia in 1 patient, and internal gaze restriction in 1 patient). The primary clinical data are summarized in Table 1 . FAST and BE-FAST scales were applied; stroke suspicion was detected in 14 (61%) patients according to the FAST scale, while stroke suspicion was detected in 17 (74%) patients according to the BE-FAST scale.

**Table 1:** Clinical characteristics and evaluation of the patients

Case	Sex/Age	Symptoms	MRI- angiography	Location	*MTHFR* 677C > T	*MTHFR* A1298C	Factor V Leiden	*PAI-1*	Other risk factors	Prognosis
1	M/3 m	Seizure/Left hemiplegia	N	Right frontoparietal	N	N	N	N	Viral Infection	Unknown
2	M/13 years	Hiccup/Hemiplegia	−	Right cerebellum	−	−	−	−	Brain tumor surgery	Discharge
3	M/2 m	Apnea/Seizure	−	Left thalamus	−	−	−	−	−	Physiotherapy
4	F/4 m	Anizocoria	Left MCA	Left occiputoparietal	Heterozygote	Heterozygote	N	4G/5G	−	Unknown
5	M/ year	Right hemiplegia	−	Left caudate nucleus	−	−	−	−	Down syndrome, Fallot's tetralogy	Discharge
6	M/17,5y	Left hemiplegia	Right PCA	Multiple locations	Heterozygote	Heterozygote	N	4G/5G	−	Physiotherapy
7	M/2 m	Apnea	Right MCA, ACA	Right frontoparietal	N	Heterozygote	N	N	−	Discharge
8	M/9 m	Right hemiplegia	Left MCA	Left parietal	N	Heterozygote	N	N	−	Physiotherapy
9	M/15 years	Diplopia	N	Left thalamus	N	Heterozygote	N	4G/5G	−	Normal
10	F/7,5 years	Headache/Facial asymmetry/Right hemiplegia	Left ICA	Left internal capsule	Homozygote	N	N	N	−	Normal
11	M/1,5 m	Seizure	Left MCA	Left Frontoparietal/Basal ganglia	−	−	−	−	Decreased C and S proteins	Unknown
12	M/3 years	Facial asymmetry	−	Right Basal ganglia	Homozygote	N	N	N	−	Normal
13	M/4 years	Left hemiplegia	N	Pons	N	Homozygote	Heterozygote	N	Hydrocephalia, VP shunt	Physiotherapy
14	F/5 years	Left hemiplegia	Right ICA	Right parietaoccipital	N	Heterozygote	N	4G/5G	Hypochondroplasia, congenital ICA hypoplasia	Normal
15	F/7 years	Right hemiplegia/Facial asymmetry	Left MCA	Left caudate nucleus	N	Heterozygote	N	N	−	Physiotherapy
16	F/7 years	Confusion	N	Left caudate nucleus	N	N	N	N	−	Normal
17	M/16,5y	Right hemiplegia	N	Left internal capsule	Homozygote	N	Heterozygote	N	−	Physiotherapy
18	F/6 years	Confusion	N	Left thalamus	−	−	−	−	Viral infection	Unknown
19	M/12 years	Left hemiplegia/Facial asymmetry	Right MCA	Right frontoparietal	N	N	N	N	Supraventricular tachycardia	Physiotherapy
20	F/1,5 m	Seizure	Right MCA	Right frontoparietal	N	N	N	N	−	Physiotherapy
21	M/5 years	Visual disturbance ataxia	−	Pons	N	Homozygote	N	N	−	Normal
22	M/9,5 years	Seizure	N	Left internal capsule	−	−	−	−	Hydrocephalia VP shunt, cerebral palsy	Physiotherapy
23	F/13 Y	Headache, facial asymmetry	N	Left caudate nucleus	N	N	N	N	−	Physiotherapy

Abbreviations: ACA, anterior cerebral artery; ICA, internal carotid artery; *MTHFR* , *methylenetetrahydrofolate reductase* gene; MRI, magnetic resonance imaging; N, Normal; MCA, middle cerebral artery; *PAI-1* , *serpin family E member 1* gene; PCA, posterior cerebral artery; VP, ventriculoperitoneal.

**Figure 1:**
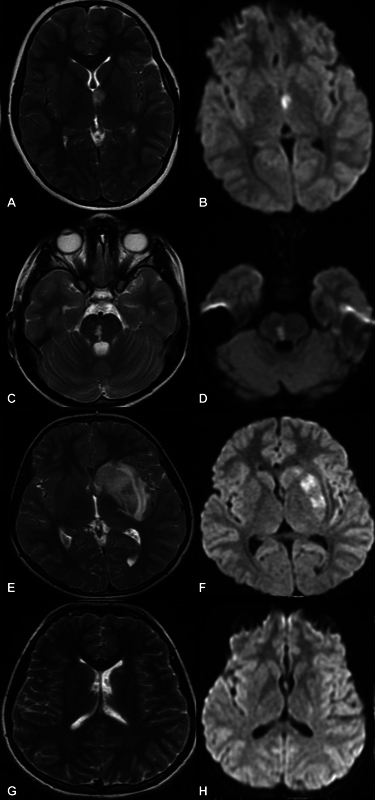
( **A** ) Case 18: axial T2-weighted (T2-W) image revealing hyperintensity in the superior part of the left anterior thalamus; ( **B** ) axial diffusion-weighted imaging (DWI) showing high intensity in the superior part of the left anterior thalamus. ( **C** ) Case 21: hyperintense lesion in the right lateral region of the pons on axial T2-W image; ( **D** ) DWI revealing high intensity in the right lateral region of the pons. ( **E** ) Case 23: T2-W image revealing hyperintense lesion surrounded with edema in the left lentiform nucleus and caudate nucleus; ( **F** ) left lentiform nucleus and caudate nucleus regions showing high signal on DWI. ( **G** ) After the treatment, small hyperintense lesion in the left laterale caudate nucleus on axial T2-W. ( **H** ) After the treatment, normal findings on DWI.

The mean time between the patients' admission to the emergency department and the time of diagnosis was of 17.3 (median: 12.5) hours. Brain CT scans were performed in 12 (52%) patients: infarction was detected in 8 (66%), and 4 (34%) had standard CT scans. We also performed MRI scans in all patients: infarction was in the cerebrum in 19 (83%) patients (13 in the left cerebrum, 6 in the right cerebrum), in the pons in 2 (9%), in the cerebellum in 1 (4.5%), and in the multiple areas in 1 patient (4.5%). In 18 (78%) patients, an MRA study was conducted, and the occlusion of the middle cerebral artery was detected in 7 of these subjects. Congenital internal carotid artery hypoplasia was detected in 1 patient with hypochondroplasia. We performed MRI spectroscopy in 1 patientdue to MRI findings causing mass effects ( Figure 1 ).

When the prothrombotic risk factors were evaluated, mutations of the *methylenetetrahydrofolate reductase* ( *MTHFR* ; A1298C and 677C > T) gene were found most frequently. The results of the genetic analysis were obtained in 18 patients (78%): an A1298C heterozygous mutation was found in 7 patients (39%), and an A1298C homozygous mutation, in 2 patients (11%). While a 677C > T heterozygous mutation was observed in 2 patients (11%), a 677C > T homozygous mutation was found in 3 subjects (17%). A heterozygous factor V Leiden mutation was noticed in 2 patients (11%), and a 4G/5G heterozygous mutation of the serpin family E member 1 ( *PAI-1* ) gene was found in 3 subjects (16%). The levels of proteins C and S were low in 1 patient (5%); 7 subjects (30%) had structural heart defects; 5 (22%) presented patent foramen ovale (PFO); 1 (4%) patient presented atrial septal defect (ASD); 1 subject was operated on for teratology of Fallot; 1 patient presented supraventricular tachycardia (SVT); 2 subjects had a recent history of viral infection; 2 patients (8%) had a ventriculoperitoneal (VP) shunt; and, lastly, 1 subject underwent surgery due to a brain tumor.

When we grouped the sample according to the TOAST classification, we found that 1 patient (4%) was in the small vessel disease group, 2 (9%), in the large vessel atherosclerosis group, 10 (43%), in the other determined etiology group, 4 (17%), in the multiple probable/possible etiology group, and 6 patients were in the undetermined etiology group.

A total of 18 patients (78%) received low-molecular-heparin treatment, while 5 (21%) did not receive any treatment. Three (13%) patients died during the hospitalization period, and 11 (47%) continued to receive physical therapy due to neurological deficits. The clinical findings of 6 patients (26%) underwent complete improvement. Since 3 patients (13%) could not be contacted, data on their latest status could not be obtained.

## DISCUSSION

Arterial ischemic stroke is defined as focal cerebral damage resulting from sudden occlusion or rupture of cerebral arteries. Not common in childhood, it is a leading cause of morbidity and mortality. 12 The diagnosis is delayed due to the heterogeneity of the etiology and risk factors, the presence of nonspecific and varying symptoms, the higher frequency of diseases in the differential diagnosis compared with stroke, and the inability to efficiently perform imaging methods in children. 5 Some studies 13 have shown that the time to diagnose AIS in children is delayed up to 23 hours. In the present study, the mean time until diagnosis was of 17.3 (median: 12.5) hours. The main reasons for the delay are heterogeneous initial symptoms, low clinical awareness, and the inability to perform diagnostic imaging methods quickly.

Hemiplegia is the most prevalent symptom of a localized neurological problem in children who have had an ischemic stroke, and AIS may change with age and present with many different symptoms in children. While apnea, drowsiness, reluctance to eat, sepsis-like findings, and focal convulsions are observed in infants, neurological findings such as hemiparesis, seizures, speech problems, visual disorders, headaches, and language difficulties (including aphasia) may be observed in older children. 14 In our study, the most common reason for hospital admission was hemiplegia. Some patients presented with rare symptoms, such as upward gaze restriction, diplopia, anisocoria, and hiccups, which rarely accompany AIS. It should not be forgotten that an infarction may present with very different symptoms depending on its location. With the application in children of tools used in adults, such as the FAST test, the BE-FAST scale, or the Recognition Stroke in the Emergency Room (ROSIER) scale, pediatric AIS cases may be easily distinguished from situations that mimic stroke. 15 We applied the FAST and BE-FAST scales; we detected suspected stroke in 14 (61%) children according to the FAST scale and in 17 (74%) children according to the BE-FAST scale. Gerstl et al. reported that when the BE-FAST scale was applied to children, 96% of the patients met the findings and that it could allow for a higher rate of stroke pre-diagnosis.

Non-contrast CT can be performed quickly in the emergency department. It can adequately exclude hemorrhagic stroke or parenchymal abnormalities. However, CT may not show signs of AIS in the early period. 16 In the current study, 34% of the patients presented normal cranial CT scans. Cranial and diffusion MRI scans were performed in all patients, and involvement was found mainly on the left side of the brain (56%). The most frequently occluded artery was the middle cerebral artery (MCA; 38%). Many studies have stated that the left side of the brain is affected most frequently. Besides, it has been shown 17 18 that there is occlusion mostly in the MCA. The differential diagnosis of ischemic stroke can include brain tumor, hemorrhagic stroke, subdural hemorrhage, drug toxicity, meningitis, or encephalitis. While cranial MRI imaging provides rapid and accurate diagnosis of stroke in many cases, in some cases MRI may be insufficient to diagnose stroke. As in case 23 of the present study, who presented mass effect, further examinations may be necessary for the diagnosis. One study 19 reported a case required a stereotaxic biopsy to differentiate the tumor.

Arterial ischemic stroke causes high economic costs and psychiatric disorders for the patients and their families due to complete or partial neurological deficits. 20 Unlike in children, conditions such as hypertension, heart failure, metabolic syndrome and coronary artery disease are risk factors for stroke in adults. In children, the most critical risk factor is congenital or acquired heart disease, which can be detected in one-third of the cases. 21 Moreover, prothrombotic predisposition, sickle cell anemia, and infections are important risk factors. 22 Abnormal cardiac anatomy is thought to increase the risk of stroke due to many complex reasons, such as paradox shunt of the embolus, prothrombotic state secondary to inflammation, iron deficiency anemia, and reduced cardiac functions. 23 Numes and Fox 24 reported that, in the International Pediatric Stroke Study (IPSS), 31% of cardiac anomalies were detected, a rate similar to the 8% found in the Kaiser Pediatric Stroke Study (KPSS). In the present study, structural heart anomalies, including ASD, Fallot's tetralogy, and PFO, were detected in 30% of the patients, and SVT was detected in 1 patient. In children, SVT is considered a potential risk factor for cardioembolic stroke. 25 The role of the PFO in AIS and the assumption that complete closure of the PFO reduces the risk of stroke recurrence is controversial. Patent foramen ovale may cause venous thrombus to reach the brain with a right-to-left shunt mechanism. 26 In the Randomized Evaluation of Recurrent Stroke Comparing PFO Closure to Established Current Standard of Care Treatment (RESPECT) study, 27 which was conducted in adults, the authors stated that closing the PFO does not cause a decrease in the risk of stroke recurrence. In previous studies, the presence of PFO was not necessarily considered a factor directly increasing the risk of stroke. However, the etiology of stroke has been associated with PFO, especially in young children with no other known cause of stroke.

Many causes of congenital and acquired thrombophilia are associated with the frequency of childhood AIS. It has been stated 28 that genetic factors play an essential role in the development of ischemia in studies involving family members and twins. The 677C > T *MTHFR* polymorphism is one of the most commonly investigated polymorphisms in adults and children; MTHFR 5 reduces methylenetetrahydrofolate to 5 methylenetetrahydrofolates, the main form of folate in plasma. The transition of a single base pair affects the thermolability of *MTHFR* , reducing its activity and increasing the homocysteine level. Thus, *MTHFR* polymorphism is thought to be a genetic risk factor in arterial ischemia. 29 In line with this hypothesis, some studies have reported a relationship between the *MTHFR* 677C > T polymorphism and AIS. Nevertheless, other studies 30 indicate no such relationship. The *MTHFR* A1298C polymorphism has also been investigated. 31 However, there is a contradiction in accepting this polymorphism as a risk factor for AIS. In a meta-analysis, Kang et al. 31 reported that the A1298C polymorphism is a significant risk factor for the development of ischemic stroke (odds ratio [OR]: 1.227; 95%CI: 1.062–1.416). 31 In the current study, mutations were not examined in 5 out of 23 patients. The genetic analyses for C1289C, 677C > T, Factor V Leiden, and plasminogen activator inhibitor-I in 18 patients. We detected A1298C heterozygous mutation in 7 (39%) patients, A1298C homozygous mutation in 2 (11%), 677C > T homozygous mutation in 3 (17%), and 677C > T heterozygous mutation in 2 (11%) patients. Similar to previous studies, *MTHFR* polymorphisms, which are accepted as factors increasing the risk of AIS, were the most commonly detected mutations in the present study. Sarecka-Huar et al., 32 in a study on the relationship of the plasminogen activator inhibitor-I mutation with AIS, evaluated that this mutation did not increase the risk of ischemia in children; the authors only detected the *PAI-I* 4G/5G polymorphism in 3 patients (17%).

Although cardiogenic factors and prothrombotic causes are the most common causes of AIS in childhood, some studies 33 have evaluated arteriopathies as an essential risk factor for childhood AIS. In the current study, congenital internal carotid artery (ICA) hypoplasia, which may be asymptomatic due to the development of collateral vascular structures, 34 was detected in 1 patient with hypochondroplasia. Arterial ischemic stroke in the patient with congenital internal carotid artery hypoplasia or hypochondroplasia has not been previously reported in the literature. The case of a 35-year-old patient with achondroplasia in whom stroke was detected due to a thrombus in the ICA has been reported. 35 In 2006, the Childhood Cancer Survivor Study 36 reported that patients who were followed and treated for intracranial tumors had 30 times the risk of developing stroke compared with their twin siblings; in this study, cerebellar infarction was detected in one patient who received radiotherapy treatment due to brain tumor. The Children's Oncology Group's Children's Oncology Group. Long-Term Follow-up Guidelines for Survivors of Childhood, Adolescence, and Young Adult Cancer 37 recommend that patients receiving radiation therapy above 18 Gray should undergo neurological examinations yearly.

Classifications such as the Childhood AIS Standardized Classification and Diagnostic Evaluation (CASCADE) and TOAST, which are used in AIS in adults, are also used in children. However, few studies on it have been conducted. In a study 38 which included 36 children and evaluated AIS cases using the TOAST classification, 80.5% of the children fell into the “other etiology identified” category; large vessel atherosclerosis or small vessel disease was not detected in any patient; and multiple probable stroke etiologies were only identified in 1 child. In the present study, we found that 1 patient (4%) fell into the “small vessel disease” category, 2 (9%), into the “large vessel atherosclerosis”, 10 (43%), into the “other determined etiology”, 4 (17%), into the “multiple probable/possible etiology” category, and 6, into the “undetermined etiology” category.

Contrary to the study we mentioned above, a high rate (4 patients (%17)) in our study was in the “multiple possible/probable etiology” category. We think that this different result in our study is due to the fact that stroke was detected in many different patients, such as VP shunt, brain tumor and achondroplasia. Many different reasons, which are yet to be fully clarified, are blamed for the etiology of stroke. Acute ischemic stroke secondary to VP shunt dysfunction, for example, has been reported in a child with Moyamoya syndrome. 39

In conclusion, AIS, which is a significant cause of morbidity and mortality, can present with very different clinical findings in childhood. Patients can present with neurological deficits that can easily suggest the diagnosis of AIS, as well as nonspecific findings such as hiccups, anisocoria, upward gaze restriction, and apnea. In the current study, we have also shown that brain CT scans taken in the early period will not exclude the diagnosis of stroke. It should be kept in mind that concomitant conditions, such as operation due to an intracranial tumor, cardiological abnormalities, and hypochondroplasia, may increase the risk of stroke. It would be appropriate to perform advanced tests such as MRA in these patients.
